# Focal adhesion kinase plays an essential role in Th17 cell differentiation by stimulating NF-κB signaling

**DOI:** 10.3389/fimmu.2025.1596802

**Published:** 2025-09-19

**Authors:** Hyeong Su Kim, Ji Hyeon Lee, Hyogon Sohn, Woo Ho Lee, Ga Eul Kim, Wonyong Lee, Hang-Rae Kim, Gap Ryol Lee

**Affiliations:** ^1^ Department of Life Science, Sogang University, Seoul, Republic of Korea; ^2^ Center for Viral Immunology, Institute for Basic Science, Daejeon, Republic of Korea; ^3^ Samsung Precision Genome Medicine Institute, Research Institute for Future Medicine, Samsung Medical Center, Seoul, Republic of Korea; ^4^ Department of Health Sciences and Technology, Samsung Advanced Institute for Health Sciences & Technology (SAIHST), Sungkyunkwan University, Seoul, Republic of Korea

**Keywords:** FAK, Th17, NF-κB, T helper differentiation, EAE

## Abstract

T helper type 17 (Th17) cells play critical roles in the pathogenesis of various autoimmune and inflammatory diseases; however, signaling pathways that affect Th17 cell differentiation are not fully understood. Here, we investigated whether focal adhesion kinase (FAK), an integrator of extracellular signals, regulates differentiation of Th17 cells. The findings reveal that *Fak* deficiency in CD4 T cells significantly reduces Th17 differentiation, while also promoting regulatory T (Treg) cell differentiation, thereby ameliorating symptoms of experimental autoimmune encephalomyelitis (EAE). Mechanistically, *Fak* deficiency inhibited nuclear translocation of the NF-κB subunit RelA, thereby reducing the binding of RelA to the promoter region of *Il17a*. Moreover, pharmacological inhibition of FAK with the specific inhibitor PND1186 prevented Th17 differentiation *in vitro*, and reduced EAE symptoms *in vivo*. Thus, FAK plays an essential role in Th17 cell differentiation by stimulating NF-κB signaling.

## Introduction

CD4 T cells play a vital role in host defense against invading pathogens. Naïve CD4 T cells are stimulated by antigen-presenting cells (APCs) presenting antigens with major histocompatibility complex II (MHCII) and provide co-stimulatory molecules and various cytokines (1). When activated, naïve CD4 T cells differentiate into different subsets, including T helper type 1 (Th1), Th2, Th17, and Treg cells (2–5). Among these, Th17 cells produce IL-17A, IL-17F, and IL-22, which play critical roles in mediating immune responses against extracellular pathogens (6, 7). However, Th17 cells contribute to various autoimmune diseases, including multiple sclerosis (MS), psoriasis, and rheumatoid arthritis (8–11). Differentiation of Th17 cells relies on cytokines such as IL-6, IL-1β, TNFα, and TGFβ. Among these, IL-6 is of particular importance because it activates STAT3 (12), eventually leading to expression of RORγT, the key lineage-determining transcription factor of Th17 cells (13).

In addition to cytokines, CD4 T cell differentiation pathways can also be affected by other signals, including the strength of the T cell receptor (TCR), binding to costimulatory molecules, various metabolic pathways, and the microbiota (5, 14). Sensing extracellular environmental cues is also crucial for regulating CD4 T cell differentiation and function. One such mediator of these signals is focal adhesion kinase (FAK, also known as PTK2), a non-receptor protein-tyrosine kinase that plays a major role in intracellular signal transduction (15). Originally identified as a protein kinase involved in mediating signals at the point of focal adhesion, FAK regulates cell migration and adhesion (15). Recent studies indicate that FAK functions as a scaffold protein, integrating various signals (16). FAK comprises three domains: the N-terminal four-point ezrin/radixin/moesin (FERM) domain, the C-terminal focal adhesion targeting (FAT) domain, and the kinase domain (17). Activation of FAK occurs when integrins bind to the extracellular matrix or endothelial cell surface, leading to phosphorylation of the Y397 residue of FAK, which is located in the linker region between the FERM and kinase domains. Phosphorylation of other residues by Src family kinases such as Src, Lck, and Fyn, further enhances FAK activity (18–20); thus, FAK integrates signals from the extracellular environment and mediates processes such as cell migration, cell adhesion, and cytoskeleton reorganization (21, 22).

Among immune cells, FAK mediates the activity of macrophages (23) and neutrophils (24). Additionally, studies have highlighted the close relationship between FAK and T cell activation (25–28). However, the role of FAK during T cell activation remains controversial, with one study reporting that FAK negatively regulates T cell activation (29), while another shows that FAK promotes it (30). A recent study also suggests that FAK mediates TCR-independent and extracellular matrix-mediated sensitization of T cell activation (28). Furthermore, a specific isotype of integrin, integrin α3, promotes Th17 cell differentiation by bolstering the immunological synapse and enhances Th17 cell migration through the blood-brain barrier during brain inflammation (31). Taken together, these results suggest that FAK may integrate signals generated by the extracellular environment, thereby affecting T cell differentiation. This led us to investigate the role of FAK during differentiation of CD4 T cells.

The NF-κB pathway is triggered by TCR signaling. Upon stimulation of CD4 T cells by APCs through the TCR, the activation signal is transduced through multiple components, ultimately leading to activation of transcription factors NF-κB (32), NFAT (33), and AP-1 (34). These factors drive proliferation and differentiation of CD4 T cells. The NF-κB pathway has two branches: canonical and noncanonical. The NF-κB family comprises five proteins: p65 (RelA), c-Rel, RelB, NFκB1 (p105/p50), and NFκB2 (p100/p52) (35). Typically, TCR signaling triggers the canonical pathway, thereby activating the p65 (or c-Rel)/p50 complex, which translocates to the nucleus and induces the expression of various genes necessary for T cell activation.

Here, we used a retrovirus-mediated Cre transduction system (*in vitro*) and Th17-specific *Fak*-deficient mice to investigate the role of FAK during Th17 cell differentiation. Our findings reveal that deletion of *Fak* inhibits differentiation of Th17 cells by blocking the NF-κB pathway. Th17-specific *Fak*-deficiency ameliorated symptoms of experimental autoimmune encephalomyelitis (EAE) and blocked differentiation of Th17 cells *in vivo*. Moreover, the specific FAK inhibitor PND1186 effectively prevented Th17 cell differentiation *in vitro*, and alleviated EAE symptoms *in vivo*. Taken together, these results strongly suggest that FAK plays a crucial role in Th17 differentiation by activating the NF-κB pathway.

## Results

### FAK is required for Th17 cell differentiation

**Figure 1:**
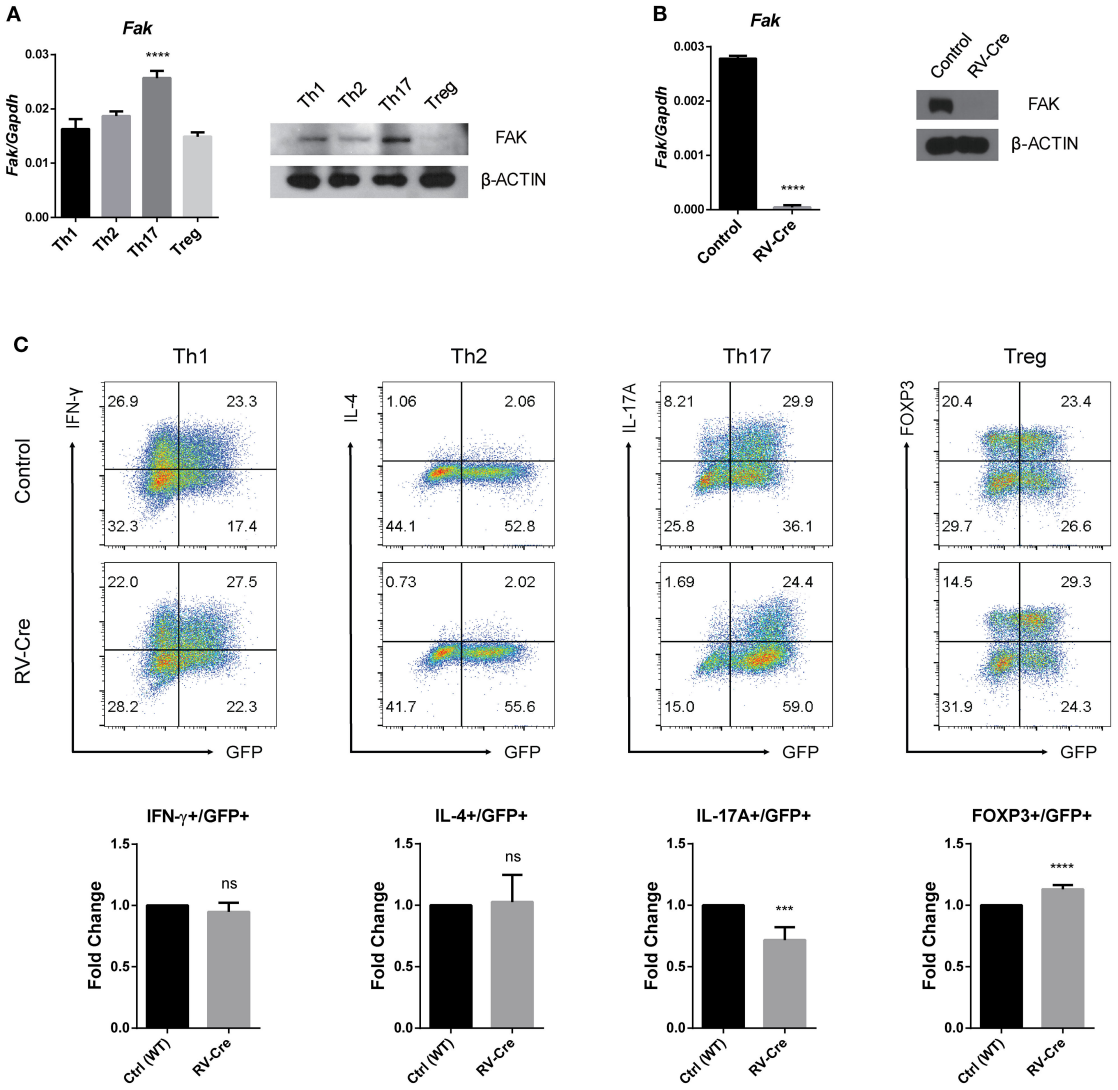
FAK is highly expressed in and required for Th17 cells. **(A)** Naïve CD4 T cells were cultured under each subset differentiation conditions for 3 days. The transcript level of *Fak* was measured by RT-qPCR (left) and the protein level of FAK was measured by immunoblot analysis (right). **(B, C)** Naïve CD4 T cells from *Fak* ^fl/fl^ mice were introduced with a control empty vector (control) or a CRE recombinase-expressing vector (RV-Cre) to induce *Fak* deletion and cultured under Th17-polarizing conditions for 3 days **(B)** or various subset-polarizing conditions **(C)** for 3 days. **(B)** GFP+ cells were sorted. The transcript level of *Fak* was measured by RT-qPCR (left) and the protein level of FAK was measured by immunoblot analysis (right). **(C)** The expression of GFP, IFN-γ, IL-4, IL-17A, and FOXP3 was analyzed by flow cytometry (top). The statistical analysis was performed on pooled data from five independent experiments (bottom). Error bars represent the standard deviation. The significance of differences between groups was determined by one-way ANOVA **(A)** and Student *t* test **(B, C)**. ***P < 0.001; ****P < 0.0001, n.s., not significant.

To investigate the role of FAK in CD4 T cells, we first measured *Fak* expression in different CD4 T cell subsets. Naïve CD4 T cells were isolated from the spleens of C57BL/6 mice and cultured for 3 days under specific subset-polarizing conditions. FAK expression was highest at both mRNA and protein levels in Th17 cells among the various subsets (**Figure 1A**). Next, we explored the effect of FAK on CD4 T cell differentiation using a gene deletion system. We isolated naïve CD4 T cells from Fak^fl/fl^ mice and introduced a Cre-expressing retroviral vector (RV-Cre) into the cells to induce gene deletion (**Figure 1B**). We examined the effect of FAK deficiency on CD4 T cell differentiation by stimulating the control or RV-Cre-transduced cells under various subset-polarizing conditions. Since RV-Cre contains a GFP-coding sequence (36), the transduced cells can be identified by fluorescence during FACS analysis. Deletion of *Fak* reduced Th17 cell differentiation and slightly increased Treg cell differentiation (**Figure 1C**). Differentiation into Th1 and Th2 cells was not affected. These results suggest that FAK specifically affects Th17 cell differentiation.

**Figure 2:**
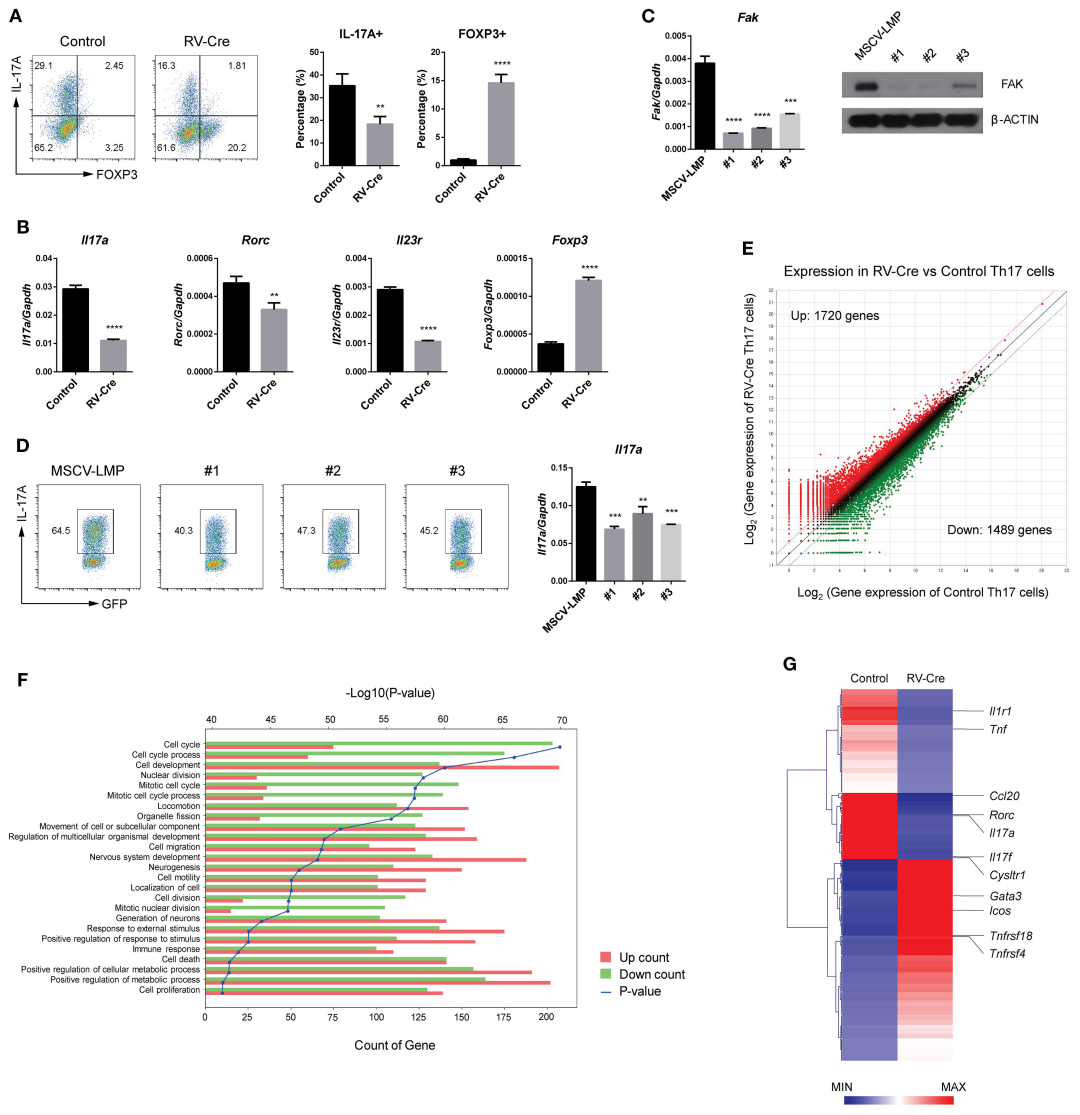
FAK affects Th17 cell differentiation program. **(A, B)** Naïve CD4 T cells from *Fak* ^fl/fl^ mice were cultured and sorted as **Figure 1B**. **(A)** IL-17A+ and FOXP3+ cells were measured by flow cytometry. **(B)** Transcript levels of *Il17a*, *Rorc*, *Il23r*, and *Foxp3* were measured by RT-qPCR. **(C, D)** Naïve CD4 T cells were introduced with either the control vector (MSCV-LMP) or *Fak* shRNA vectors (#1, #2, and #3) and cultured under Th17-polarizing conditions for 3 days. **(C)** The transcript level of *Fak* was measured by RT-qPCR (left) and protein level of FAK was measured by immunoblot analysis (right). **(D)** IL-17A+ cells among the vector-transduced cells (GFP+) were measured by flow cytometry (left). GFP+ cells were sorted and the transcript level of *Il17a* was measured by RT-qPCR (right). All of RT-qPCR data were normalized to *Gapdh*. **(E–G)** Naïve CD4 T cells were transduced with control or RV-Cre and cultured under Th17-polarizing conditions for 3 days. GFP+ cells were sorted and subjected to RNA-seq analysis. **(E)** Scatter plot of RNA-seq data. **(F)** Gene ontology analysis of differentially expressed genes (DEGs) from control and RV-Cre-transduced Th17 cells. **(G)** Heatmap of immune/inflammatory response-related genes among the DEGs from control and RV-Cre-transduced Th17 cells. All of RT-qPCR data were normalized to *Gapdh*. Data in **(A–D)** are pooled from three independent experiments. Error bars represent the standard deviation. The significance of differences between groups was determined by Student *t* test. **P < 0.01; ***P < 0.001; ****P < 0.0001.

To further investigate the role of FAK in Th17 and Treg cell differentiation, we measured subset-specific genes in sorted GFP+ cells from control and RV-Cre-transduced cells using flow cytometry and reverse transcription quantitative polymerase chain reaction (RT-qPCR). *Fak*-deficiency reduced expression of IL-17A, while that of FOXP3 increased markedly in Th17 cells (**Figures 2A, B**). The transcript levels of *Rorc* and *Il23r* also decreased in RV-Cre-transduced Th17 cells (**Figure 2B**). Additionally, to examine whether FAK has the same effect on pathogenic Th17 cells, we stimulated RV-Cre-transduced CD4 T cells under pathogenic Th17-polarizing conditions and measured expression of *Il17a* and *Foxp3* (**Supplementary Figure S1**). Consistent with data from conventional Th17 cells, *Fak*-deficient pathogenic Th17 cells showed reduced expression of *Il17a*, but increased expression of *Foxp3*. To further confirm the effects of FAK in Th17 cells, we used an shRNA-mediated knockdown method. ShRNA-mediated knockdown of FAK (**Figure 2C**) reduced expression of *Il17a* (**Figure 2D**), but increased that of *Foxp3* (**Supplementary Figure S2**). Taken together, these data indicate that FAK is required for Th17 cell differentiation *in vitro*.

Next, we conducted RNA-sequencing (RNA-seq) to identify altered genes in RV-Cre-transduced (GFP+) Th17 cells (**Figure 2E** and GEO accession # GSE298541). Expression of 1,720 genes increased, while that of 1,489 genes decreased, in RV-Cre-transduced Th17 cells compared with control Th17 cells (**Figure 2E**). Gene ontology analysis revealed significant changes in multiple biological processes in RV-Cre-transduced Th17 cells, including “cell cycle”, “cell development”, “nuclear division”, “locomotion”, and “immune response” (**Figure 2F**). Moreover, differentially expressed gene analysis, focusing on genes related to immune or inflammatory responses (**Figure 2G**), revealed that expression of Th17-related genes such as *Il17a*, *Il17f*, *Rorc*, and *Cysltr1* (37) by RV-Cre-transduced Th17 cells decreased, while that of Treg-related genes such as *Tnfrsf18* and *Tnfrsf4* increased. Taken together, these data strongly suggest that FAK is expressed at high levels by Th17 cells, and essential for expression of signature genes associated with Th17 cells.

### *Fak* deficiency ameliorates the severity of EAE

**Figure 3:**
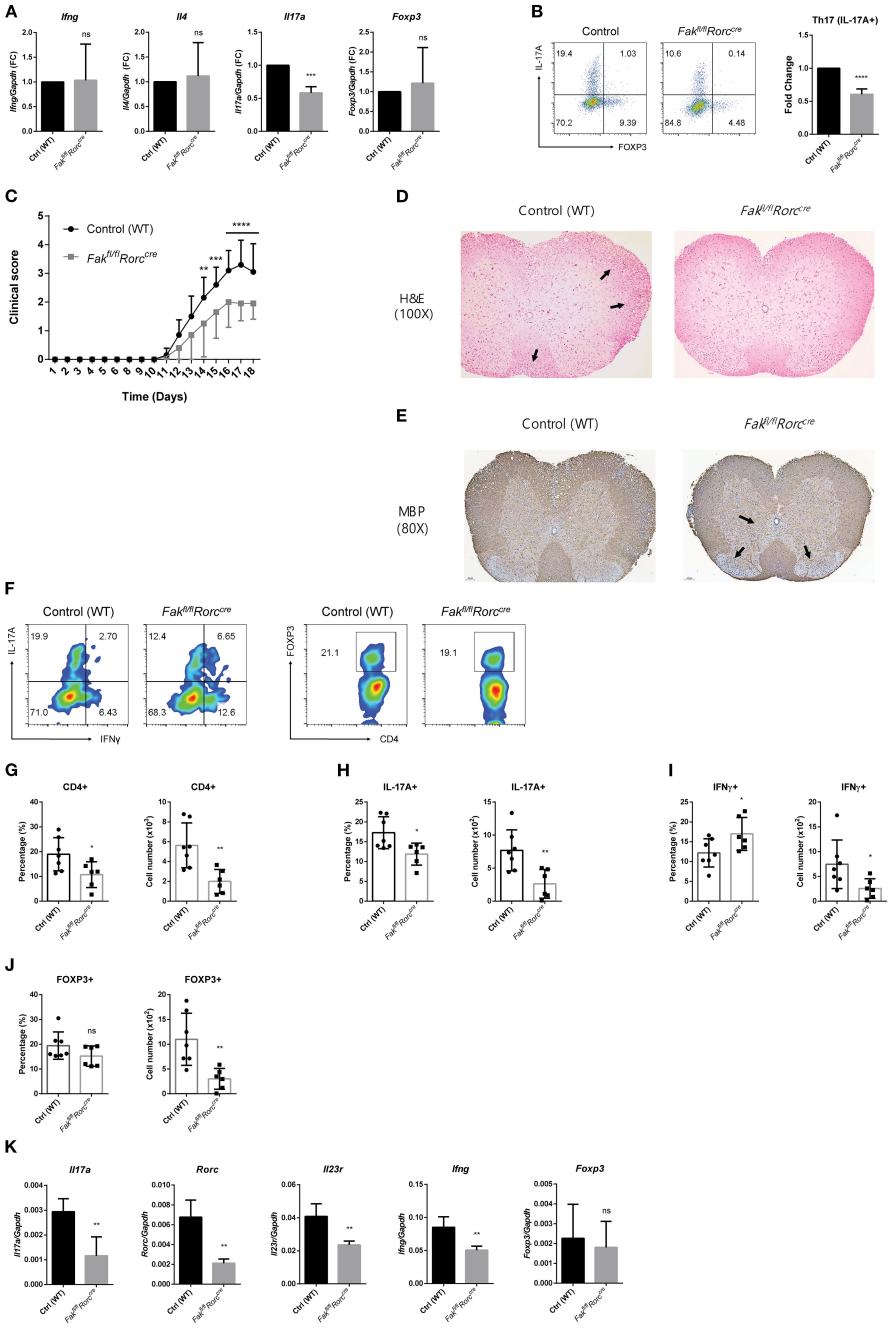
FAK deficiency ameliorates the severity of EAE. **(A, B)** Naïve CD4 T cells from WT and *Fak^fl/fl^Rorc^cre^* mice were cultured under polarizing conditions toward each different subset for 3 days. Transcript levels of *Ifng* for Th1 cells, *Il4* for Th2 cells, *Il17a* for Th17 cells, and *Foxp3* for Treg cells were measured by RT-qPCR **(A)** and the percentage of IL-17A+ cells under Th17-polarizing conditions was measured by flow cytometry **(B)**. **(C)** EAE was induced in control (WT, n = 10) and *Fak^fl/fl^Rorc^cre^* (n = 10) mice as described in the Materials and Methods section. The symptoms of EAE were monitored every day and clinical scores were evaluated after EAE induction. **(D, E)** Histopathological analysis of lumbar spinal cords from control and *Fak^fl/fl^Rorc^cre^* mice at the peak of the disease. **(D)** H&E stained sections of spinal cords. Arrows indicate the inflammatory foci. **(E)** Immunohistochemical staining of myelin basic protein (MBP). Arrows indicate the more preserved myelin in *Fak^fl/fl^Rorc^cre^* mice compared with control mice. **(F)** IL-17A+ and IFNγ+ cells (left) and FOXP3+ cells (right) among CNS-infiltrating CD4 T cells were measured by flow cytometry. **(G–J)** The percentage and absolute number of CNS-infiltrating mononuclear cells were measured. **(G)** CD4+ cells, **(H)** IL-17A+ cells, **(I)** IFNγ+ cells, and **(J)** FOXP3+ cells. **(K)** Transcript levels of *Il17a*, *Rorc*, *Il23r*, *Ifng*, and *Foxp3* in CNS-infiltrating mononuclear cells were measured by RT-qPCR. Data were normalized to *Gapdh*. Data in **(F–K)** are pooled from ten independent experiments. Error bars represent the standard deviation. The significance of differences between groups was determined by Student *t* test. *P < 0.05; **P < 0.01; ***P < 0.001; ****P < 0.0001, n.s., not significant.

To further investigate the role of FAK *in vivo*, we generated *Fak* conditional KO mice by crossing Fak^fl/fl^ mice with transgenic mice expressing Cre recombinase under the control of the *Rorc* promoter (hereafter referred to as *Fak^fl/fl^Rorc^cre^* mice). To assess the efficiency of *Fak* deletion in Th17 cells, we measured *Fak* mRNA expression across each polarized CD4 T cell subset. *Fak* expression was greatly reduced in Th17 cells, while it remained intact in other subsets (**Supplementary Figure S3**). Naïve CD4 T cells from WT and *Fak^fl/fl^Rorc^cre^* mice were stimulated under various polarizing conditions and their gene expression was measured by RT-qPCR. Expression of subset-specific markers by Th1 (*Ifng*), Th2 (*Il4*), and Treg (*Foxp3*) cells remained intact, regardless of *Fak* deletion (**Figure 3A**). Importantly, the level of *Il17a* mRNA (**Figure 3A**), and the percentage of IL-17A+ cells (**Figure 3B**), fell markedly in the *Fak^fl/fl^Rorc^cre^* Th17 cell population compared with the control (WT) Th17 cell population.

Next, we induced EAE, a Th17-driven model of MS (38, 39), by immunizing both WT control and *Fak^fl/fl^Rorc^cre^* mice with myelin oligodendrocyte glycoprotein (MOG35-55) peptide plus pertussis toxin. Clinical scores revealed that *Fak* deficiency attenuated autoimmune-related symptoms (**Figure 3C**). Histological examination of spinal cords using hematoxylin and eosin (H&E) staining and myelin basic protein (MBP) immunohistochemistry revealed significantly less inflammatory cell infiltration and reduced myelin damage in *Fak^fl/fl^Rorc^cre^* mice (**Figures 3D, E**). Flow cytometry analysis of CD4 T cells infiltrating the central nervous system (CNS) (spinal cord) of control and *Fak^fl/fl^Rorc^cre^* mice revealed that the percentage of IL-17A+ cells was lower in the CNS of *Fak^fl/fl^Rorc^cre^* mice (**Figure 3F**), while the percentage of FOXP3+ cells remained unchanged (**Figure 3F**). Furthermore, the percentage and number of CD4 cells and IL-17+ cells were reduced in the CNS-infiltrating mononuclear cells from *Fak^fl/fl^Rorc^cre^* mice compared with the control mice (**Figures 3G, H**). The percentage of IFNγ+ cells slightly increased but their number decreased (**Figure 3I**). The percentage of Foxp3+ cells remained unchanged, but their number decreased (**Figure 3J**). We also measured expression of various signature genes in CNS-infiltrating mononuclear cells by RT-qPCR. The findings revealed that cells from *Fak^fl/fl^Rorc^cre^* mice showed lower expression of Th17 signature genes such as *Il17a*, *Rorc*, and *Il23r*, as well as the Th1-related gene *Ifng*, with no alteration in expression of *Foxp3* (**Figure 3K**). Collectively, these data demonstrate that FAK plays a critical role in an animal model of Th17-mediated autoimmune diseases.

### FAK regulates the STAT3 signaling pathway in Th17 cells

**Figure 4:**
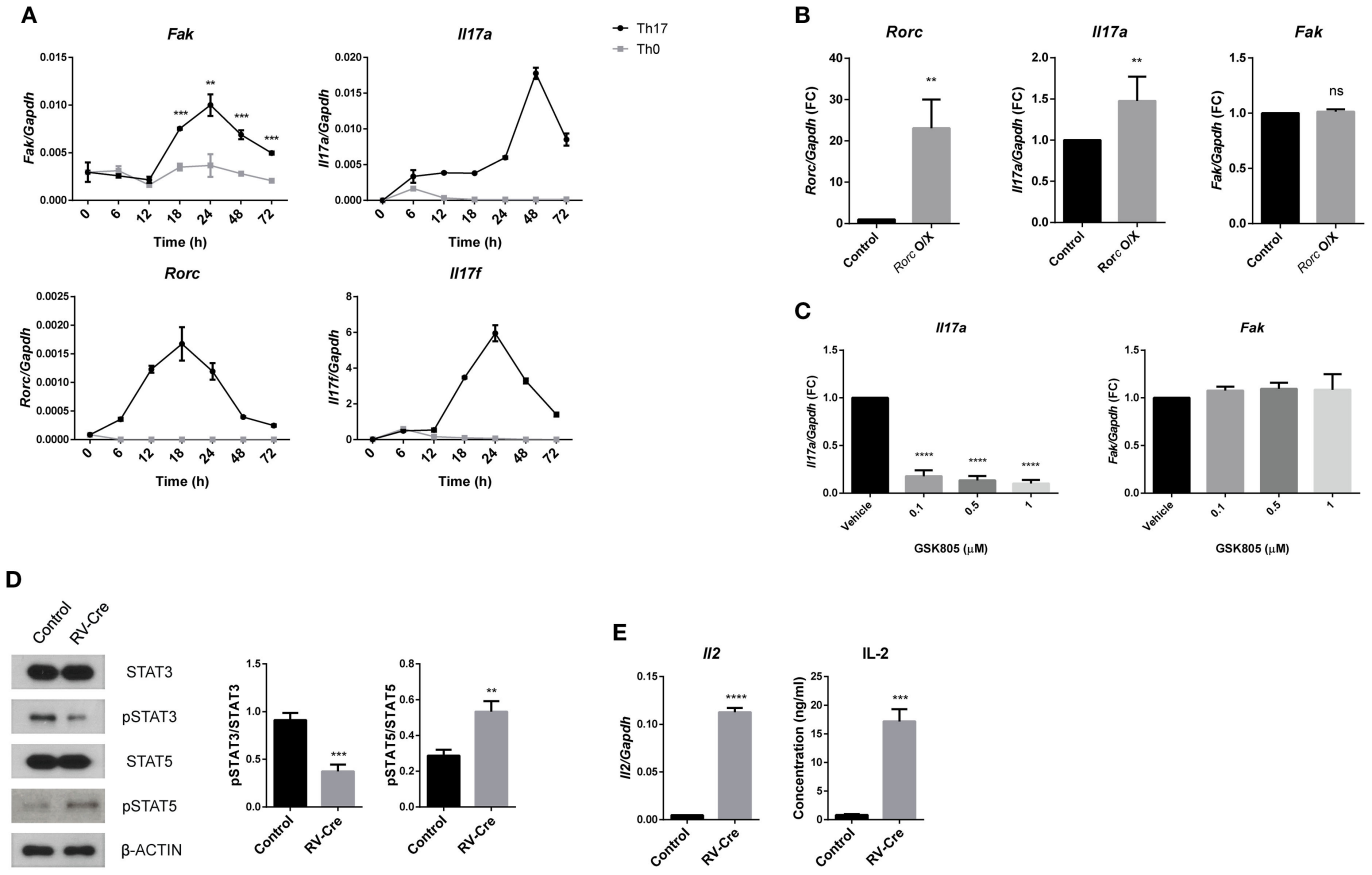
FAK regulates the STAT3 signaling pathway in Th17 cells. **(A)** Naïve CD4 T cells were cultured under various differentiation conditions for the indicated time periods and the transcript levels of *Fak*, *Il17a*, *Rorc*, and *Il17f* were measured by RT-qPCR. **(B)** Naïve CD4 T cells were introduced with control vector or *Rorc*-expressing vector (*Rorc* O/X) and cultured in Th17-polarizing conditions for 3 days. Transcript levels of *Rorc*, *Il17a*, and *Fak* were measured by RT-qPCR. **(C)** Naïve CD4 T cells were cultured under Th17-polarizing conditions with dose-dependent treatment of GSK805 for 3 days. Transcript levels of *Il17a* and *Fak* were measured by RT-qPCR. **(D, E)** Naïve CD4 T cells from *Fak* ^fl/fl^ mice were cultured as described in **Figure 1B** and GFP+ cells were sorted. **(D)** Each protein level was measured by immunoblot analysis (left) and the ratios of pSTAT3/STAT3 and pSTAT5/STAT5 were calculated by densitometry (right). **(E)** Transcript level of *Il2* was measured by RT-qPCR (left) and the protein level of IL-2 from the supernatants was measured by ELISA (right). All of RT-qPCR data were normalized to *Gapdh*. Data in **(A, B, D, E)** were pooled from three independent experiments, and data in **(C)** from five. Error bars represent the standard deviation. The significance of differences between groups was determined by Student *t* test. **P < 0.01; ***P < 0.001; ****P < 0.0001, n.s., not significant.

Next, to assess the time-dependent kinetics of *Fak* expression in Th17 cells, we cultured naïve CD4 T cells for 3 days under Th17-polarizing conditions and harvested them at different time points. We then measured the levels of *Fak* transcripts, as well as those of Th17 signature genes, using Th0 cells as a control (**Figure 4A**). *Fak* expression was induced after 18 h of cultivation, and was highest at 24 h. Expression of *Rorc*, *Il17f*, and *Il17a* peaked at 18, 24, and 48 h, respectively. The level of *Rorc* transcripts peaked at 18 h, followed by *Il17f*, *Il17a*, and *Fak*. RORγT (encoded by *Rorc*), induced during the early differentiation stage, regulates expression of Th17 signature genes such as *Il17a*, *Il17f*, and *Il23r* (13). To test whether FAK expression is regulated by RORγT, we introduced a *Rorc*-expressing vector into Th17 cells (**Figure 4B**). Ectopic expression of *Rorc* increased *Il17a* expression but had no effect on *Fak* expression in Th17 cells. We then assessed the effect of RORγT functional inhibition using GSK805, a well-characterized RORγT inhibitor (40). Consistent with a previous report (40), GSK805 markedly suppressed *Il17a* expression, whereas *Fak* expression remained unchanged (**Figure 4C**). These results suggest that FAK is not a direct downstream target of RORγT.

STAT3 is a key signaling component for Th17 cell differentiation, and is activated by IL-6 (12). Once activated, STAT3 forms a homodimer with another STAT3 molecule and translocates into the nucleus to induce expression of Th17 signature genes. Typically, activation of STAT3 is triggered by JAK, located downstream of the IL-6 receptor (41); however, recent studies revealed that STAT3 can also be activated by direct interaction with FAK, in lymphatic endothelial cells and cancer cells (42–44). To assess whether FAK affects activation of STAT3 in Th17 cells, we measured pSTAT3 levels in *Fak*-deficient Th17 cells. *Fak*-deficient Th17 cells showed decreased levels of pSTAT3, but increased levels of pSTAT5 (**Figure 4D**). As STAT3 and STAT5 act reciprocally, and IL-2 is a key cytokine required for STAT5 activation (45, 46), we next examined expression of *Il2* in *Fak*-deficient Th17 cells to determine whether FAK affects the reciprocal roles of STAT3 and STAT5 (**Figure 4E**). *Il2* expression was elevated in *Fak*-deficient Th17 cells, along with pSTAT5 levels. Taken together, these data indicate that *Fak*-deficiency reduces activation of STAT3, but induces phosphorylation of STAT5 leading to IL-2 cytokine production by Th17 cells.

### FAK regulates Th17 cell differentiation through the NF-κB pathway

**Figure 5:**
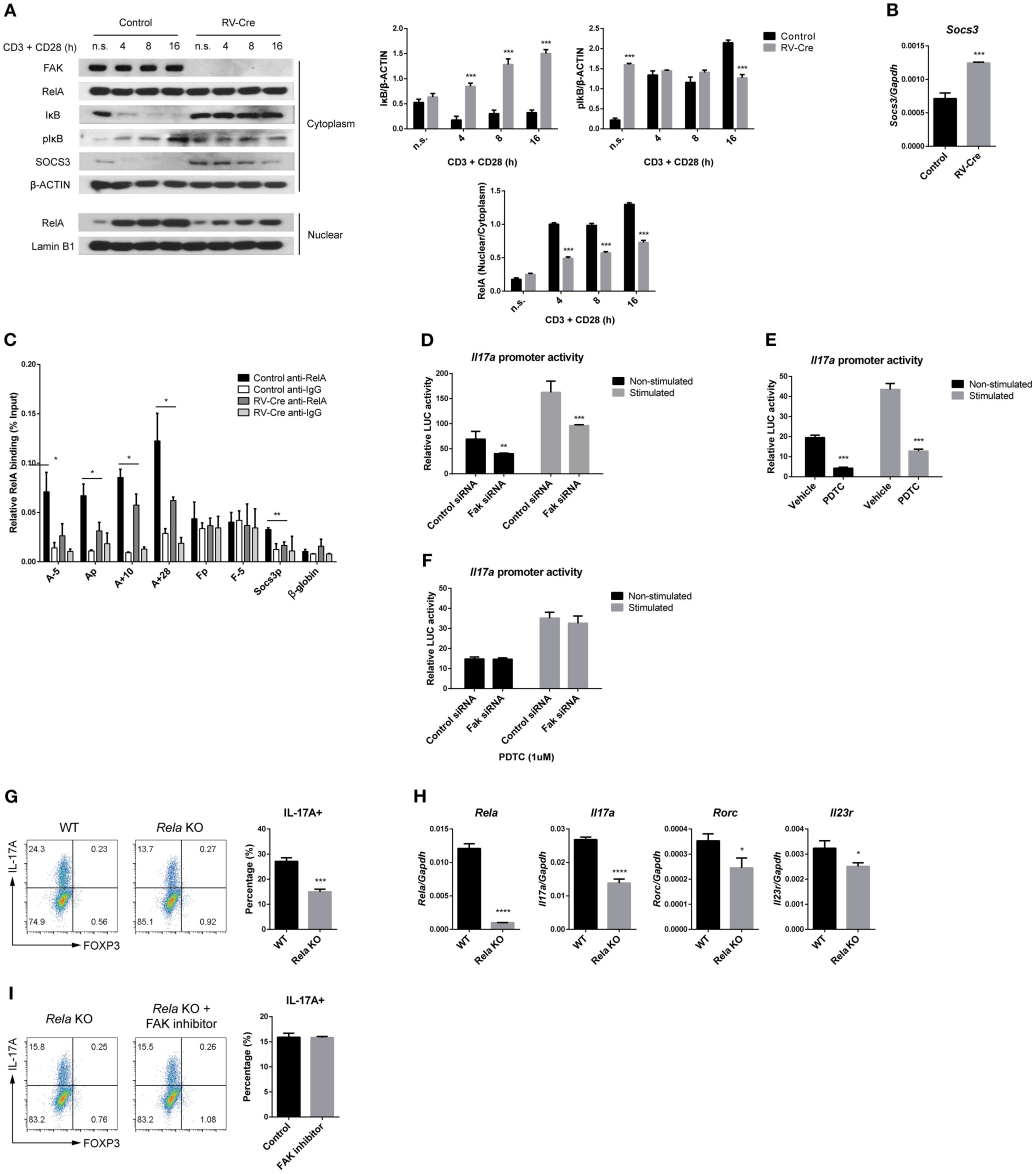
FAK regulates Th17 cell differentiation through the NF-κB pathway. **(A)** Naïve CD4 T cells from *Fak* ^fl/fl^ mice were transduced with control vector or RV-Cre and cultured under Th17-polarizing conditions for 3 days. GFP+ cells were sorted and rested in normal media for 2 days and serum-free medium for an additional 8 hours. The cells were restimulated with anti-CD3/CD28 antibodies for the indicated time periods, and nuclear/cytoplasmic extracts were prepared. Each protein level was measured by immunoblot analysis (right). The ratios of IκB/β-Actin, pIκB/β-Actin, and nuclear RelA/cytoplasmic RelA were calculated by densitometry (right). **(B, C)** Naïve CD4 T cells from *Fak* ^fl/fl^ mice were transduced with RV-Cre and cultured under Th17-polarizing conditions for 3 days **(B)** and 16 hours **(C)** and GFP+ cells were sorted. **(B)** Transcript level of *Socs3* was measured by RT-qPCR. **(C)** Relative RelA binding to each indicated locus was measured through ChIP assay. Nuclear extracts from cultured cells were reacted with an anti-RelA antibody, and precipitated DNA fragments were measured by qPCR. Isotype-matching IgG was used as a negative control. **(D–F)** *Il17a* promoter activity was measured through luciferase assay. **(D)** EL4 cells were transfected with pGL3-*Il17a* promoter vector and control or *Fak* siRNA, and the cells were rested for 20 hours. After transfection, the cells were divided into non-stimulated or stimulated groups, with the stimulated groups received 4 hour stimulation with PMA/ionomycin. **(E)** EL4 cells were transfected with the pGL3-*Il17a* promoter vector and rested for 20 hours with or without PDTC treatment (1 μM). **(F)** EL4 cells were transfected as described in **(D)** and rested for 20 hours with 1 μM PDTC treatment. **(G–I)** Naïve CD4 T cells from *Rela* ^fl/fl^ mice were introduced with a control empty vector (WT) or a Cre recombinase expressing vector (p65 KO) to induce *Rela* deletion and cultured under Th17-polarizing conditions for 3 days. **(G)** IL-17A+ and FOXP3+ cells were measured by flow cytometry. **(H)** GFP+ cells were sorted and transcript level of *Rela*, *Il17a*, *Rorc*, and *Il23r* were measured by RT-qPCR. **(I)** Naïve CD4 T cells from *Rela* ^fl/fl^ mice were cultured as described in **(G)** and additionally treated with vehicle (control) or FAK inhibitor (PND1186, 1 μM). IL-17A+ and FOXP3+ cells were measured by flow cytometry. RT-qPCR data in **(B, H)** were normalized to *Gapdh*. Data in **(A–I)** are pooled from three independent experiments. Error bars represent the standard deviation. The significance of differences between groups was determined by Student *t* test. *P < 0.05; **P < 0.01; ***P < 0.001; ****P < 0.0001.

The NF-κB pathway plays a crucial role in various biological processes in CD4 T cells, including survival, maintenance, and differentiation (47). In a resting state, NF-κB is located in the cytosol, where it is bound and masked by the inhibitor of NF-κB (IκB). Upon stimulation, IκB kinase (IKK) becomes activated and phosphorylates IκB, triggering its degradation. The released NF-κB then translocates to the nucleus and activates the expression of its target genes. Notably, NF-κB serves as a direct substrate for FAK (48), and FAK regulates activation of the NF-κB pathway in non-immune cells (49, 50). Based on this knowledge, we formulated a hypothesis that FAK might affect Th17 cell differentiation through the NF-κB pathway. To test the hypothesis, we first examined the effect of *Fak* deficiency on the NF-κB pathway in Th17 cells. Upon stimulation with anti-CD3/CD28, the NF-κB pathway was activated in WT Th17 cells, but not in *Fak*-deficient Th17 cells (**Figure 5A**). Notably, cytosolic IκB levels did not change, and translocation of RelA into the nucleus decreased, in *Fak*-deficient Th17 cells compared with WT Th17 cells. Additionally, phosphorylation and degradation of IκB were downregulated (**Figure 5A**). It is noteworthy that the protein SOCS3 inhibits both the JAK/STAT (51, 52) and NF-κB signaling pathways (53) by promoting protein degradation. Expression of SOCS3 at both the protein (**Figure 5A**) and RNA (**Figure 5B**) levels increased in *Fak*-deficient Th17 cells, suggesting disruption of both the STAT3 and NF-κB signaling pathways.

Several STAT3 and NF-κB binding sites have been identified in the *Il17a* promoter and the intergenic regions between the *Il17a* and *Il17f* genes (54, 55). These binding sites play a crucial role in transcriptional regulation of *Il17a* expression in response to various stimuli. Therefore, we conducted chromatin immunoprecipitation (ChIP) assays to investigate whether FAK affects binding of RelA to the regulatory regions of the *Il17a* and *Il17f* genes. We observed RelA binding to the promoter and distal regulatory regions of the *Il17a* gene including -5kb, +10kb, and +28kb regions (56, 57), and the promoter and +5kb enhancer region of the *Il17f* gene in Th17 cells (**Figure 5C**); however, deletion of *Fak* led to a significant reduction in RelA binding to the *Il17a* promoter, whereas binding to the *Il17f* promoter remained unaltered (**Figure 5C**). Additionally, binding of RelA to the promoter of *Socs3* was also inhibited in *Fak*-deficient Th17 cells (**Figure 5C**). These results suggest that FAK regulates RelA binding to the *Il17a* and *Socs3* genes.

Next, to investigate the effect of FAK on activity of the *Il17a* promoter, we conducted a transient reporter assay. EL4 mouse thymoma cells were transfected with reporter constructs containing the *Il17a* promoter, with or without *Fak* siRNA, and then treated with phorbol 12-myristate 13-acetate (PMA) and ionomycin (**Figure 5D**). The results showed that the promoter activity of *Il17a* was significantly reduced upon siRNA-mediated knockdown of FAK expression (**Figure 5D**). A similar effect was observed after treatment with an NF-κB inhibitor (**Figure 5E**). To further explore the relationship between FAK and NF-κB during activation of the *Il17a* promoter, FAK-knockdown Th17 cells were exposed to an NF-κB inhibitor. Interestingly, the NF-κB inhibitor did not have any additional effects on reducing the activity of the *Il17a* promoter, suggesting that FAK and NF-κB are part of the same signaling pathway that regulates *Il17a* promoter activation (**Figure 5F**). To validate these findings, we used *Rela*-deficient cells. Naïve CD4 T cells were isolated from Rela^fl/fl^ mice and transduced with RV-Cre under Th17-polarizing conditions. The resulting *Rela*-deficient Th17 cells exhibited a significant reduction in IL-17A expression at both the protein (**Figure 5G**) and mRNA levels (**Figure 5H**). Expression of Th17-related genes *Rorc* and *Il23*r also decreased (**Figure 5H**). However, treatment with a specific FAK inhibitor PND1186, a pyridine reversible inhibitor of FAK (58), did not alter expression of IL-17A in *Rela*-deficient Th17 cells (**Figure 5I**). In summary, these results suggest that FAK plays a critical role in Th17 cell differentiation by facilitating recruitment of RelA, an NF-κB subunit, to the *Il17a* promoter and distal regulatory regions and stimulating the promoter activity.

### FAK inhibitors block differentiation of Th17 cells *in vitro*

**Figure 6:**
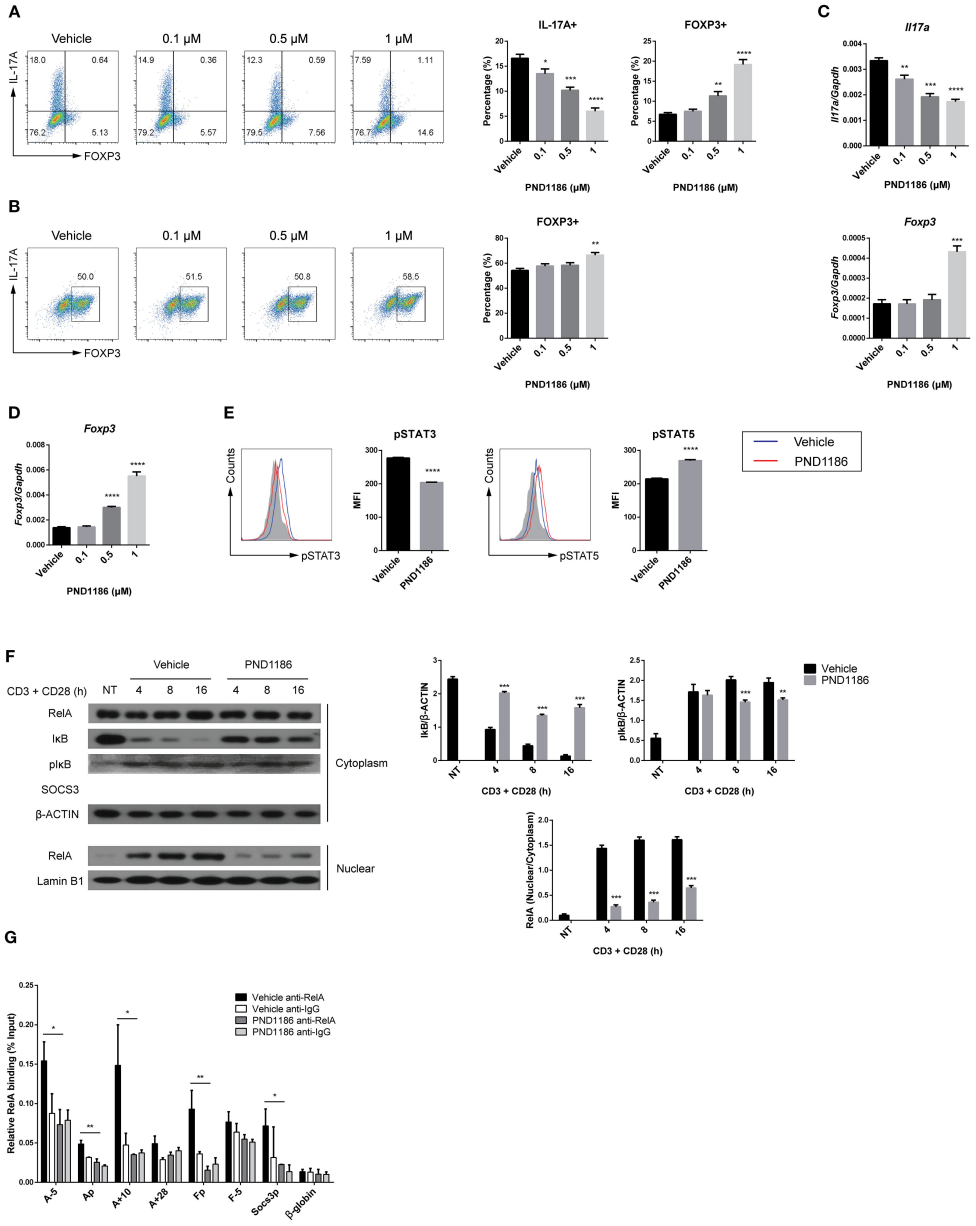
A FAK inhibitor blocks differentiation of Th17 cells *in vitro.* **(A–E)** Naïve CD4 T cells were cultured under Th17- or Treg-polarizing conditions with dose-dependent treatment of PND1186 for 3 days. IL-17A+ and FOXP3+ cells were measured by flow cytometry **(A)** and transcript levels of *Il17a* and *Foxp3* were measured by RT-qPCR **(C)** in Th17 cells. FOXP3+ cells were measured by flow cytometry **(B)** and transcript level of *Foxp3* was measured by RT-qPCR **(D)** in Treg cells. **(E–G)** Naïve CD4 T cells were cultured under Th17-polarizing conditions with vehicle (control) or PND1186 (1 μM) treatment for 3 days **(E, G)** and the indicated time periods **(F)**. **(E)** pSTAT3 and pSTAT5 levels were measured by flow cytometry. **(F)** Nuclear or cytoplasmic extracts were prepared from cultured cells, and each protein level was measured by immunoblot analysis (right). The ratios of IκB/β-Actin, pIκB/β-Actin, and nuclear RelA/cytoplasmic RelA were calculated by densitometry (right). **(G)** Relative RelA binding to each indicated locus was measured through ChIP assay. Nuclear extracts from cultured cells were incubated with an anti-RelA antibody and the precipitated DNA fragments were measured by qPCR. An isotype-matching IgG was used as a negative control. RT-qPCR data in **(C, D)** were normalized to *Gapdh*. Data in **(A–G)** are pooled from three independent experiments. Error bars represent the standard deviation. The significance of differences between groups was determined by Student *t* test. *P < 0.05; **P < 0.01; ***P < 0.001; ****P < 0.0001.

The above results suggest that FAK regulates differentiation of Th17 cells, as well as Th17-mediated disorders. Therefore, we investigated whether FAK inhibitors prevent or treat diseases associated with Th17 cells. To assess the effect of FAK inhibitors on Th17 cell differentiation *in vitro*, we cultured naïve CD4 T cells under Th17-polarizing conditions in the presence of varying concentrations of PND1186. Flow cytometry analysis revealed a dose-dependent reduction in expression of IL-17A, accompanied by an increase in FOXP3 expression (**Figure 6A**). Additionally, Treg cells generated by culturing naïve CD4 T cells with PND1186 exhibited a slight increase in the FOXP3+ cell population (**Figure 6B**). The dose-dependent changes were confirmed by RT-qPCR to detect *Il17a* and *Foxp3* transcripts (**Figure 6C**). The increase of *Foxp3* mRNA levels was more pronounced than that in the percentage of FOXP3+ cell population (**Figure 6D**). To evaluate whether PND1186 affects Th17 cell death or proliferation, we performed Annexin V and 7-AAD staining to assess apoptosis and measured the Ki-67 expression level as an indicator of proliferative activity. Although a slight increase in apoptosis was observed at the highest concentration, overall cell death was comparable to that of the control (**Supplementary Figure S4A**). Therefore, the reduction in IL-17A+ cell frequency is unlikely to be due to apoptosis. Analysis of Ki-67 mean fluorescence intensity revealed only slightly reduced proliferation of Th17 cells across all inhibitor-treated conditions, suggesting that the effect of PND1186 on Th17 differentiation is not due to its influence on cell proliferation (**Supplementary Figure S4B**). These findings suggest that PND1186 modulates the balance between Th17 and Treg cells. To validate these results, we utilized an independent FAK inhibitor, GSK2256098 (59). Treatment with GSK2256098 also led to a dose-dependent reduction in IL-17A expression at both the protein and transcript levels under Th17-polarizing conditions, and an increase in FOXP3 expression under Treg-polarizing conditions (**Supplementary Figure S5**). Consistent with the effects observed in *Fak*-deficient cells (**Figure 4D**), treatment with PND1186 significantly reduced the amount of pSTAT3, but increased that of pSTAT5, in Th17 cells (**Figure 6E**). Moreover, PND1186 reduced IκB phosphorylation and degradation, thereby inhibiting nuclear translocation of RelA (**Figure 6F**), as well as binding of RelA to the *Il17a* and *Il17f* promoters and distal regulatory regions (**Figure 6G**). Collectively, these data indicate that specific FAK inhibitors downregulate the STAT3 and NF-κB signaling pathways, thereby repressing differentiation of Th17 cells *in vitro*.

### *In vivo* administration of a FAK inhibitor attenuates the symptoms of EAE

**Figure 7:**
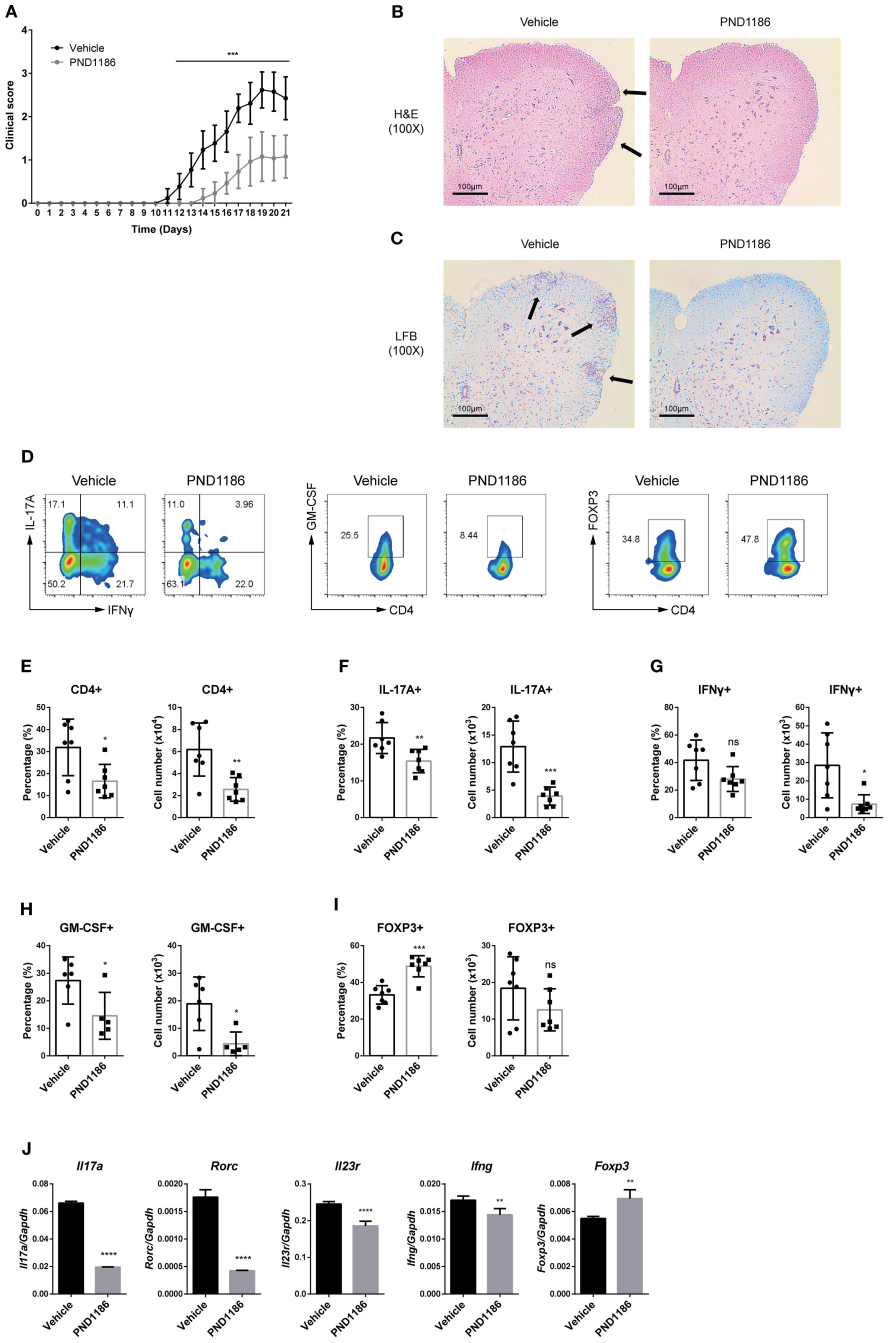
*In vivo* administration of a FAK inhibitor attenuates the symptoms of EAE. EAE was induced in C57BL/6 mice as described in the Materials and Methods section. Vehicle (control, n = 13) or PND1186 (50 mg/kg, n = 13) was administered every day from day 3 until the end of the experiments. **(A)** The symptoms of EAE were monitored daily, and clinical scores were evaluated after EAE induction. **(B, C)** Histopathological analysis of lumbar spinal cords from control or PND1186-treated mice at the peak of the disease. **(B)** H&E stained sections of spinal cords. Arrows indicate the inflammatory foci. **(C)** Luxol fast blue stained sections of spinal cords. Arrows indicate the demyelinated foci. **(D)** IL-17A+ and IFNγ+ cells (left), GM-CSF+ cells (middle), and FOXP3+ cells (right) among CNS-infiltrating CD4 T cells were measured by flow cytometry. **(E–I)** The percentage and absolute number of CNS-infiltrated mononuclear cells were measured. **(E)** CD4+ cells, **(F)** IL-17A+ cells, **(G)** IFNγ+ cells, **(H)** GM-CSF+ cells, and **(I)** FOXP3+ cells. **(J)** Transcript levels of *Il17a*, *Rorc*, *Il23r*, *Ifng*, and *Foxp3* in CNS-infiltrating mononuclear cells were measured by RT-qPCR. Data were normalized to *Gapdh*. Data in **(E–J)** are pooled from seven independent experiments. Error bars represent the standard deviation. The significance of differences between groups was determined by Student *t* test. *P < 0.05; **P < 0.01; ***P < 0.001; ****P < 0.0001, n.s., not significant.

To investigate the ability of a selective FAK inhibitor to inhibit the development of autoimmune diseases *in vivo*, we conducted experiments using an EAE model. EAE was induced in C57BL/6 mice, which were then treated daily with PND1186 (intraperitoneal injection; 50 mg/kg) or vehicle control. Remarkably, PND1186 protected mice from EAE, leading to a dramatic delay in disease onset and a notable reduction in disease severity (**Figure 7A**). Histological analysis with H&E and Luxol fast blue staining revealed a marked reduction in inflammatory infiltration and demyelination in the spinal cord of PND1186-treated mice (**Figures 7B, C**). Flow cytometry analysis of the CNS-infiltrating (spinal cord) CD4 T cells revealed a remarkable decrease in the percentage and number of IL-17A+ cells and GM-CSF+ cells, but a slight increase in those of Foxp3+ cells, in PND1186-treated mice compared with those in control mice (**Figure 7D**). Furthermore, administration of PND1186 reduced the percentage and number of CD4 cells, IL-17A+ cells, IFNγ+ cells, and GM-CSF+ cells among CNS-infiltrating mononuclear cells (**Figures 7E–H**). The percentage, but not the number, of Treg cells in the CNS of PND1186-treated mice was higher than that in vehicle controls (**Figure 7I**). Additionally, PND1186 significantly reduced the ratio and number of CNS-infiltrating granulocytes, while the number of CD8 cells and macrophages fell only slightly (**Supplementary Figure S6**). Consistent with the flow cytometry data, CNS-infiltrating mononuclear cells in PND1186-treated mice exhibited markedly lower levels of *Il17a*, *Rorc*, *Il23r*, and *Ifng* than those in control mice (**Figure 7J**). Conversely, these cells expressed higher level of *Foxp3* mRNA than control cells (**Figure 7J**). Taken together, these results indicate that the selective FAK inhibitor limits progression of EAE by suppressing Th17 differentiation.

## Discussion

Here, we used genetic, molecular biological, and pharmacological methods to demonstrate that FAK, an integrator of extracellular environmental signals, acts as a key regulator of Th17 cell differentiation. The findings suggest that lack of *Fak* reduces expression of IL-17A, which is mediated by inhibition of the STAT3 and NF-κB pathways. Deficiency of *Fak* in the EAE model resulted in a marked reduction in Th17 cell numbers in the CNS, along with reduced EAE severity. Moreover, a pharmacological inhibitor of FAK effectively suppressed development of mouse Th17 cells both *in vitro* and *in vivo*. This suggests that FAK plays an essential role in Th17 cell differentiation by activating the STAT3 and NF-κB pathways.

In this study, we investigated the role of FAK during Th17 cell differentiation. The data highlight that FAK is a signaling molecule playing an essential role in Th17 cell differentiation by stimulating NF-κB pathway. Besides their role in focal adhesion, FAK serves as a scaffold integrating various signals (16). During T cell stimulation, interactions between TCR and FAK are facilitated by direct binding of FAK to Lck/Fyn and/or CD4 (60). Upon TCR activation in human CD4 T cells, several critical tyrosine residues within FAK, including Y397, Y567/577, and Y925, become phosphorylated (30). Additionally, FAK stimulates the PI3K/Akt and MAPK/ERK pathways, which are involved in survival, proliferation, and differentiation of some cancer cells (61). Furthermore, some studies have reported that FAK leads to specific phosphorylation of IκB kinase α (IKKα) (48). We also observed a decrease in the level of phosphorylated IκB in *Fak*-deficient T cells. Therefore, it seems that FAK is involved in a variety of signaling pathways in a context-dependent manner.

Beyond cell differentiation, FAK is involved in cell migration, adhesion, and mechanosensing (16, 62, 63). Consequently, it is plausible that some of these processes impact Th17 cell function *in vivo*. Previous studies demonstrate the involvement of FAK in the interaction between APCs and T cells. Adhesion receptors such as VLA-4, LFA-1, and CD44 promote contact between T cells and APCs or target cells in secondary lymphoid tissues or at inflammatory sites. These receptors also facilitate T cell activation (64, 65). Notably, activation of the TCR, as well as LFA-1 or VLA-4, induces TCR-mediated phosphorylation of tyrosine in FAK (66, 67). Additionally, CD44 physically associates with FAK in T lymphocytes, and its activation further enhances FAK activity (68). Moreover, studies show that inhibition of FAK phosphorylation correlates with impaired TCR-induced LFA-1 clustering and adhesion to ICAM-1 in Jurkat cells (69). *Fak*-deficient CD4 T cells exhibit slightly reduced adhesion to ICAM-1, leading to weakened T cell conjugation with APCs (70). In *Fak*-deficient Th17 cells, it is possible that they form fewer immunological synapses with APCs, resulting in a lack of effective priming. Further research is needed to fully elucidate the precise mechanisms by which FAK regulates Th17 cell function.

Recruitment of circulating lymphocytes to inflammatory tissues involves complex molecular interactions with the local vascular endothelium via cell adhesion molecules and chemokines (71). Chemokine receptor signaling activates integrins on the T cell surface, enabling T cells to adhere to and migrate across the vascular wall. Among the crucial integrins involved in this process are LFA-1, VLA-4 (α4β1), and α4β7. These integrins bind to their ligands ICAM-1 and VCAM-1 on the endothelium, thereby facilitating lymphocyte transmigration (71, 72). At focal adhesions, FAK localizes and binds to the cytoplasmic tails of integrins. Consequently, FAK catalyzes several downstream signals that regulate cell adhesion and migration (73, 74). Therefore, deletion of the *Fak* gene from the Th17 cells of EAE mice might affect the capacity of Th17 cells to migrate into the CNS. This, in turn, could contribute to a lower frequency and number of Th17 cells infiltrating the CNS, leading to the observed amelioration of EAE symptoms in our study. Similarly, inhibiting FAK signaling results in reduced neutrophil transmigration (24), and impairs the motility of macrophages (75). Therefore, the FAK inhibitor PND1186 may also inhibit migratory activity, not only of IL-17A-producing cells but also of other types of immune cell. This observation could potentially explain the greater effect observed after treatment with PND1186 compared to Th17-specific deletion of *Fak* in the EAE mouse model.

To determine whether FAK is regulated by RORγT, we altered RORγT activity through both ectopic overexpression and pharmacological inhibition. Under both conditions, *Fak* expression remained unchanged, indicating that FAK is not a direct downstream target of RORγT. To explore the functional role of FAK during Th17 differentiation, we generated *Fak* conditional KO mice by crossing Fak^fl/fl^ mice with *Rorc*-Cre mice. Although this system results in delayed *Fak* deletion, occurring only after *Rorc* expression is initiated, naive CD4 T cells isolated from these mice exhibited impaired Th17 differentiation. This finding suggests that FAK plays a functionally important role, at least, at the later stages of Th17 commitment. Importantly, even after RORγT expression initiates during the early stages of Th17 differentiation, the subsequent loss of FAK can still hinder the differentiation process. Collectively, these results support the conclusion that FAK is an essential signaling mediator for IL-17A induction and proper Th17 cell differentiation.

STAT3 is critical for the activation and stabilization of RORγT expression, while NF-κB supports the inflammatory milieu required for Th17 physiology and also aids in the transcription of Th17-related genes (3, 76). Both STAT3 and NF-κB are pivotal in the differentiation and function of Th17 cells. In our study, both FAK-deficient cells and FAK inhibitors affected STAT3 and NF-κB activity. Thus, FAK actively stimulates both pathways to enhance Th17 differentiation. FAK expression, however, was not induced by RORγT. Thus, it is plausible to suggest that RORγT and FAK are independent regulators of Th17 cell differentiation, although they may interact or crosstalk at the level of STAT3 and NF-κB. In our results, FAK reduced the amount of SOCS3 protein. Additionally, FAK-stimulated NF-κB bound to the *Socs3* promoter, inhibiting the *Socs3* gene expression. Since SOCS3 is a negative regulator of STAT3 (51), it is plausible to hypothesize that this reduction in SOCS3 leads to increased STAT3 activity, suggesting a possible link between NF-κB and STAT3.

PND1186 (also known as VS 4718 or SR 2156) is a reversible, ATP-competitive FAK inhibitor with an *in vitro* IC_50_ of 1.5 nM. Selectivity profiling via Millipore’s KinaseProfiler Service revealed that 0.1 μM PND1186 inhibited FAK potently and also showed activity against FLT3, but did not significantly affect c-Src or p130Cas phosphorylation in adherent cells (58). Importantly, FLT3 is primarily expressed in hematopoietic stem and progenitor cells and is rapidly downregulated upon T-lineage commitment. Mature CD4 T cells, including Th17 cells, do not express detectable levels of FLT3, as supported by publicly available transcriptomic datasets (e.g., ImmGen). Therefore, we believe that off-target inhibition of FLT3 by PND1186 is less likely to contribute to the effects observed in our Th17 differentiation assays.

To summarize, the absence of FAK hinders differentiation of Th17 cells. This study offers crucial insights into the role of FAK during Th17 responses and in immune-related disorders. Targeting FAK as a treatment for Th17-mediated autoimmunity holds promise as a potential clinical approach, and as such, warrants further investigation.

## Materials and methods

### Mice

Female C57BL/6 mice (6–8 weeks old) were purchased from Daehan Bio Link. *Fak* ^fl/fl^ (77), and *Rela* ^fl/fl^ (78) mice were purchased from the Jackson Laboratory. All animal experiments were approved by the Sogang University Institutional Animal Care and Use Committee (approval no. IACUCSGU2019_09).

### Culture of CD4 T cells

Naïve CD4 T cells were isolated from 6–8-week-old female mice using a Mojosort™ mouse naïve CD4 T cell isolation kit (BioLegend). Purified naïve CD4 T cells were then activated by plate-bound anti-CD3ϵ (10 μg/ml) and soluble anti-CD28 (5 μg/ml) antibodies. The following antibodies and recombinant cytokines were used to differentiate each cell subset. Th1: mouse recombinant IL-2 (1 ng/ml, eBioscience), mouse recombinant IL-12 p70 (3.5 ng/ml, eBioscience), and anti-mouse IL-4 (5 μg/ml); Th2: mouse recombinant IL-2 (1 ng/ml), mouse recombinant IL-4 (5 ng/ml, R&D systems), and anti-mouse IFNγ (5 μg/ml); Th17: mouse recombinant IL-6 (50 ng/ml, R&D systems), human recombinant TGFβ1 (1 ng/ml, R&D systems), mouse recombinant TNFα (1 ng/ml, eBioscience), mouse recombinant IL-1β (2 ng/ml, Gibco), anti-mouse IFNγ (5 μg/ml), and anti-mouse IL-4 (5 μg/ml); Tregs: mouse recombinant IL-2 (1 ng/ml), human recombinant TGFβ1 (5 ng/ml), anti-mouse IFNγ (10 μg/ml), and anti-mouse IL-4 (10 μg/ml). For pathogenic Th17 cell differentiation, naïve CD4 T cells were cultured with mouse recombinant IL-6 (50 ng/ml), mouse recombinant IL-1β (2 ng/ml), mouse recombinant IL-23 (5 ng/ml, Biolegend), anti-mouse IFNγ (5 μg/ml), and anti-mouse IL-4 (5 μg/ml). PND1186, GSK2256098, and GSK805 were purchased from Selleck Chemicals and administered at the indicated dosages. All inhibitors were dissolved in DMSO, which served as the vehicle control in all experiments.

### Flow cytometry

Cells were harvested and restimulated for 4 h with the eBioscience™ cell stimulation cocktail (eBioscience). The stimulated cells were then fixed and permeabilized (eBioscience) prior to staining in accordance with the manufacturer’s instructions. Briefly, cells were stained with PerCP/Cy5.5-conjugated anti-IL-17A (BioLegend) and FITC-conjugated anti-FOXP3 (eBioscience) antibodies. For phospho flow cytometry, cells were stimulated for 3 days with Dynabeads™ mouse T-activator CD3/CD28 (Thermo Fisher Scientific) and fixed and permeabilized in fixation buffer (BioLegend), followed by staining with FITC-conjugated anti-pSTAT3 (BD Biosciences) and PerCP/eFluor 710-conjugated anti-pSTAT5 (eBioscience) antibodies. Stained cells were analyzed using Accuri C6 Plus flow cytometer (BD Biosciences). Data were analyzed with Flowjo software.

### Retroviral transduction

A total of 1.8 X 10^6^ Phoenix Eco cells were transfected with control MIEG3 (WT) or the RV-Cre retroviral plasmid (36) (a gift from Chen Dong) (2 μg) and the pCL-Eco helper plasmid (1 μg) as previously described (79). Naïve CD4 T cells were activated with plate-bound anti-CD3 and soluble anti-CD28 for 20–24 h. The T cell culture medium was replaced with virus-containing medium (i.e. supernatants from Phoenix Eco cells) supplemented with polybrene (4 μg/ml, Sigma Aldrich) and the culture plates were spun down (1600 x g, 90 min, RT) to facilitate infection. The cells were cultured under appropriate polarizing conditions for 3 days. GFP+ cells were sorted using FACSAria III (BD Bioscience) or FACSAria Fusion (BD Bioscience) and further analyzed.

### Fak knockdown assay

Fak-shRNA plasmids were cloned into an MSCV-LMP empty vector (GE Healthcare) in accordance with the manufacturer’s instructions. The following 97-mer nucleotides were used as templates: shRNA #1: 5’-TGCTGTTGACAGTGAGCGATGTCTTCAAATGATTGTGTAATAGTGAAGCCACAGATGTATTACACAATCATTTGAAGACACTGCCTACTGCCTCGGA-3’; shRNA #2: 5’-TGCTGTTGACAGTGAGCGACTACTTGATGTTATTGATCAATAGTGAAGCCACAGATGTATTGATCAATAACATCAAGTAGGTGCCTACTGCCTCGGA-3’; and shRNA #3: 5’-TGCTGTTGACAGTGAGCGCACTCAAACAGTGAAGACAAAGTAGTGAAGCCACAGATGTACTTTGTCTTCACTGTTTGAGTATGCCTACTGCCTCGGA-3’. Each shRNA was transduced into CD4 T cells using the retroviral transduction method as described above.

### Preparation of cell lysates and western blot analysis

Cells were harvested, washed with ice-cold PBS, and lysed at 4°C for 15 min with RIPA buffer (Sigma Aldrich) containing protease inhibitor (GenDEPOT). To prepare cytosolic and nucleic extracts, cells were harvested, washed, and lysed at 4°C for 10 min with hypotonic buffer containing protease inhibitor. After spinning down the cell suspension, the supernatants were collected for use as cytosolic extracts. The remaining pellets were suspended with RIPA buffer and incubated at 4°C for 15 min. These were used as nucleic extracts. Each extract was mixed with a 5X loading buffer (Thermo Fisher Scientific) and boiled for 5 min. Western blot analysis was performed as described (80). The following antibodies were used: anti-FAK, anti-p65, anti-IκB, anti-pIκB, anti-STAT3, anti-pSTAT3, anti-STAT5, anti-pSTAT5 (all from Cell Signaling Technology), anti-β-Actin (Santa Cruz Biotechnology), anti-SOCS3 (Santa Cruz Biotechnology), and anti-LaminB (Abcam).

### Total RNA isolation and RT-qPCR

Cells were harvested, homogenized in TRI reagent (Molecular Research Center), and total RNA was isolated according to the manufacturer’s instructions. Reverse transcription was performed using TOPscript RT (Enzynomics), and RT-qPCR was performed using TOPreal™ qPCR 2X PreMIX (Enzynomics) for SYBR Green, a TaqMan probe, and a LightCycler 96 (Roche). The primer sequences are listed in **Supplementary Table S1**.

### Chromatin immunoprecipitation assay

Cells (3.0 × 10^6^) were harvested, fixed with 1% formaldehyde solution for 10 min at room temperature, and then subjected to a ChIP assay using the Magna ChIP™ A/G chromatin immunoprecipitation kit (Merck Millipore) according to the manufacturer’s instructions. Cell extracts were immunoprecipitated with either an anti-RelA or normal rabbit IgG (Cell Signaling Technology) antibodies. RT-qPCR was performed using eluted DNA. The primer sequences are listed in **Supplementary Table S2**.

### Dual luciferase assay

The EL4 cells were transfected by electroporation with a pGL3-*Il17a* promoter vector, a pRL *Renilla* luciferase reporter vector, and either control or *Fak* siRNA (Santa Cruz Biotechnology). After transfection, the cells were incubated for 20 h in complete medium. On the following day, cells were stimulated for 4 h with PMA (50 ng/ml) and ionomycin (1 μM). Luciferase activity was measured using a dual-luciferase reporter assay system (Promega). Relative luciferase activity was calculated by dividing the activity of *Firefly* luciferase by that of *Renilla* luciferase.

### Induction and analysis of EAE

On Day 0, EAE was induced in female C57BL/6 mice (8–10 weeks old) using an EAE induction kit (Hooke Laboratories). For treatment with PND1186, mice were injected intraperitoneally with inhibitor or vehicle solution (50 mg/kg/day) starting from Day 3. Mice were monitored daily to measure the clinical score, which was assessed as follows: 0: no symptoms; 1: limp tail; 2: weakness of hind legs; 3: complete paralysis of hind legs; 4: complete hind leg and partial front leg paralysis; 5: moribund state. CNS-infiltrating cells were isolated as described previously (81). Isolated mononuclear cells were stimulated for 4 h with eBioscience™ cell stimulation cocktail, and then stained with the following antibodies: FITC-conjugated anti-FOXP3 (eBioscience), FITC-conjugated anti-GM-CSF, PE-conjugated anti-IFNγ, PerCP/Cy5.5-conjugated anti-IL-17A, APC-conjugated anti-CD4, FITC-conjugated anti-CD8α, PE-conjugated anti-CD11b, and PerCP/Cy5.5-conjugated anti-GR-1 (all from BioLegend), and APC-conjugated anti-CD45 antibody. Additionally, some of the isolated spinal cords were subjected to H&E and luxol fast blue staining.

### Statistical analysis

Data are presented as the mean ± standard deviation. Statistical differences between the groups were analyzed using the Student *t* test. P values < 0.05 were considered statistically significant (*P < 0.05; **P < 0.01; ***P < 0.001; ****P<0.0001).

## Data Availability

The data presented in the study are deposited in the NCBI GEO repository (accession number GSE298541).
