# Supplementary material for: Focal adhesion kinase plays an essential role in Th17 cell differentiation by stimulating NF-κB signaling
Source: Front Immunol. 2025 Sep 19;16:1596802. doi: 10.3389/fimmu.2025.1596802 (PMC12491285; doi:10.3389/fimmu.2025.1596802)
Supplement: Supplementary file 1 [file DataSheet1.docx]

**Supplementary figures and tables**

Figure S1. *Fak* deficiency causes inhibition of differentiation of both conventional and pathogenic Th17 cells.

Figure S2. ShRNA-mediated knockdown of FAK increased expression of *Foxp3*.

Figure S3. Specific deletion of Fak in Th17 cells using Fak^fl/fl^Rorc^cre^ mice.

Figure S4. FAK inhibitor PND1186 had minimal effects on cell death and proliferation in Th17 cells.

Figure S5. FAK inhibitor GSK2256098 reduces IL-17A expression in Th17 cells, but enhances FOXP3 expression in Treg cells.

Figure S6. PND1186 has various effects on CNS-infiltrating granulocytes, CD8 T cells, and macrophages.

Table S1. Primer list used for RT-qPCR

Table S2. Primer list used for ChIP assay


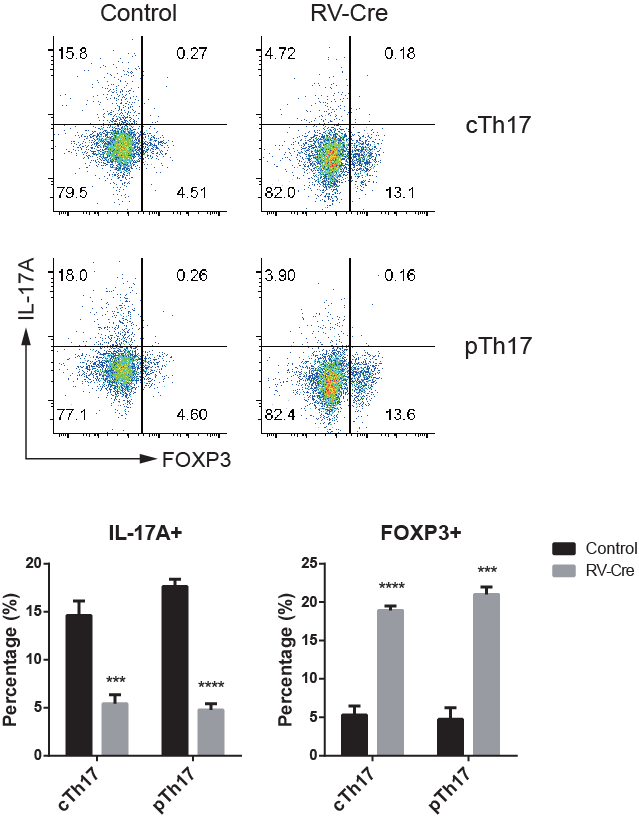


**Figure S1. *Fak* deficiency causes inhibition of differentiation of both conventional and pathogenic Th17 cells.**

Naïve CD4 T cells from *Fak*^fl/fl^ mice were introduced with either a control empty vector (Control) or a Cre recombinase expressing vector (RV-Cre) to induce *Fak* deletion and cultured under conventional Th17- or pathogenic Th17-polarizing conditions for 3 days. GFP+ cells were sorted and IL-17A+ and FOXP3+ cells were measured by flow cytometry. Data were pooled from three independent experiments (bottom). Error bars represent the standard deviation. The significance of differences between groups was determined by Student *t* test. ***, P < 0.001; ****, P < 0.0001.


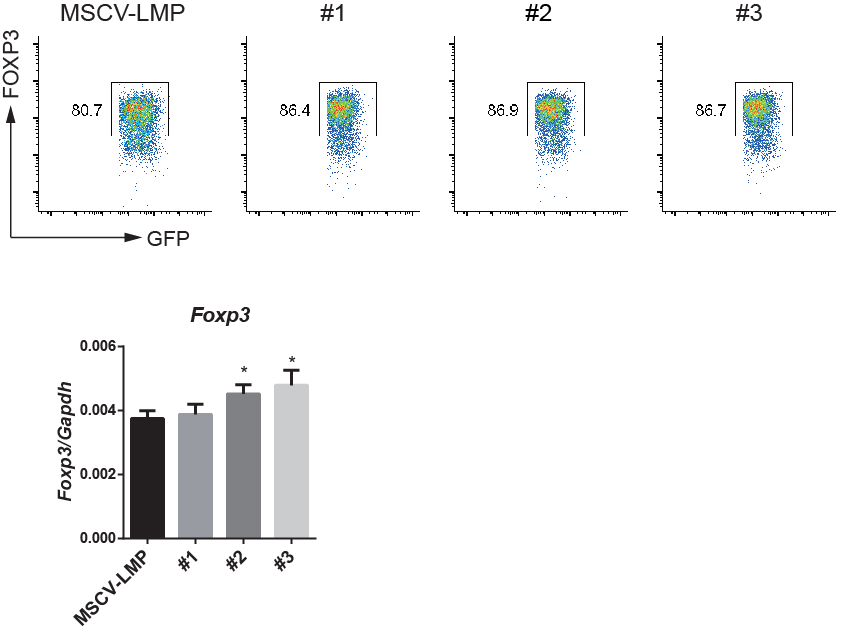


**Figure S2. ShRNA-mediated knockdown of FAK increased expression of *Foxp3*.**

Naïve CD4 T cells were introduced with either the control vector (MSCV-LMP) or *Fak* shRNA vectors (#1, #2, and #3) and cultured under Treg-polarizing conditions for 3 days. FOXP3+ cells among the vector transduced cells (GFP+) were measured by flow cytometry (left). GFP+ cells were sorted, and the transcript level of *Foxp3* was measured by RT-qPCR (right). Data were normalized to *Gapdh* and pooled from three independent experiments. Error bars represent the standard deviation. The significance of differences between groups was determined by Student *t* test. *, P < 0.05.


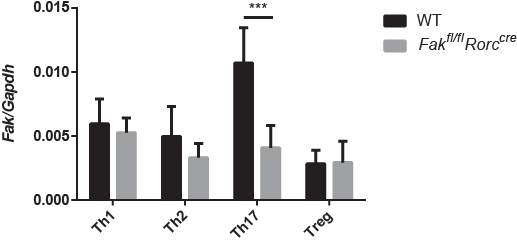


**Figure S3. Specific deletion of Fak in Th17 cells using *Fak*^fl/fl^*Rorc*^cre^ mice**.

Naïve CD4 T cells from WT and *Fak^f^*^l/fl^*Rorc*^cre^ mice were cultured under various polarizing conditions for 3 days. Transcript levels of *Fak* were measured by RT-qPCR. Data normalized to *Gapdh* expression were pooled from four individual experiments. Error bars represent the standard deviation. The significance of differences between groups was determined by two-way ANOVA. ***, P < 0.001.


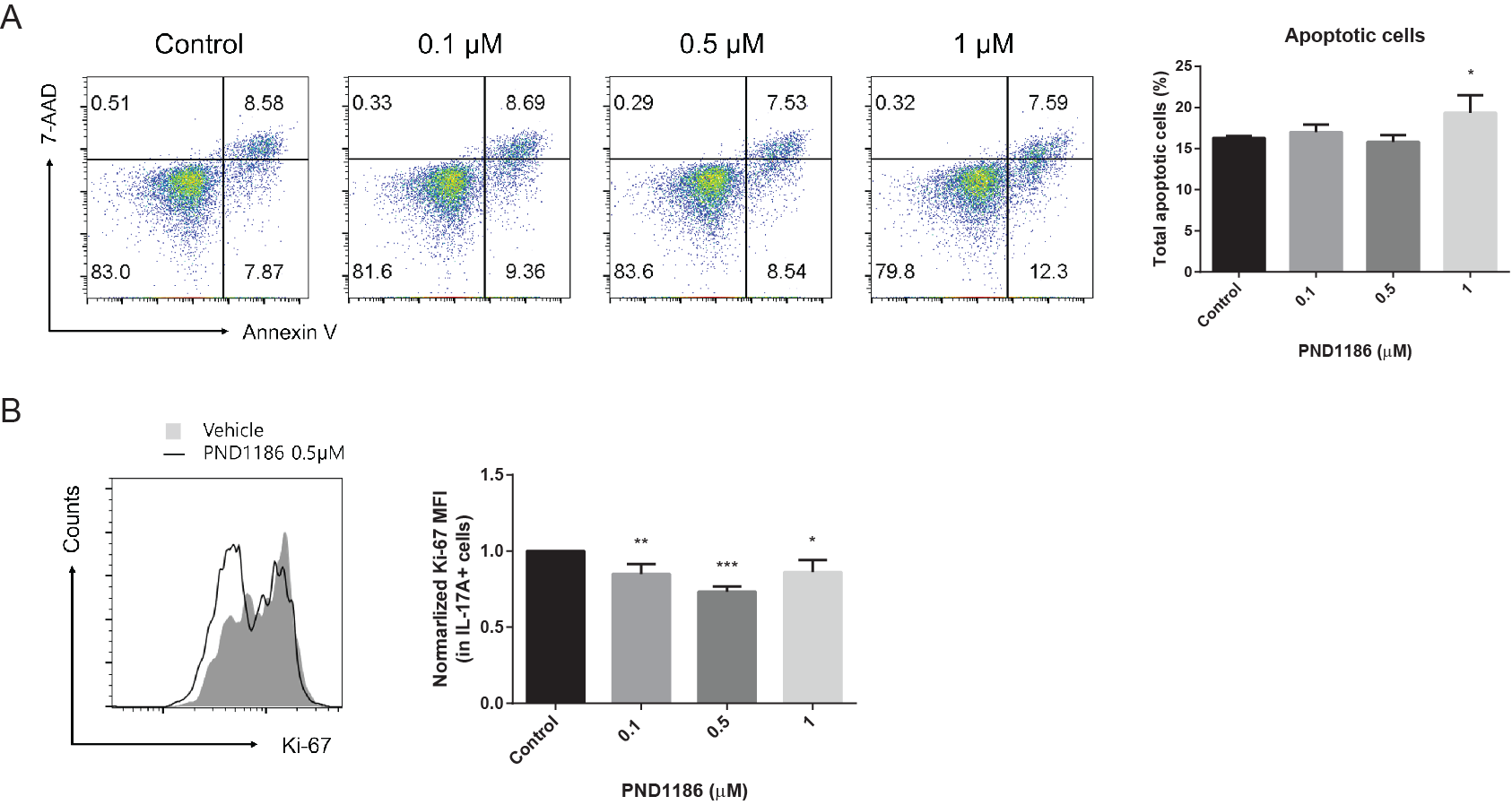


**Figure S4. FAK inhibitor PND1186 had minimal effects on cell death and proliferation in Th17 cells.**

(A and B) Naïve CD4 T cells were cultured under Th17 polarizing conditions with dose-dependent treatment of PND1186 for 3 days. Annexin V+ and 7-AAD+ cells were measured by flow cytometry (A). Mean fluorescence intensity (MFI) of Ki-67 was determined by flow cytometry (B). Data in A are pooled from three individual experiments, and data in B are from four. Error bars represent the standard deviation. The significance of differences between groups was determined by Student *t* test. *, P < 0.05; **, P < 0.01; ***, P < 0.001.


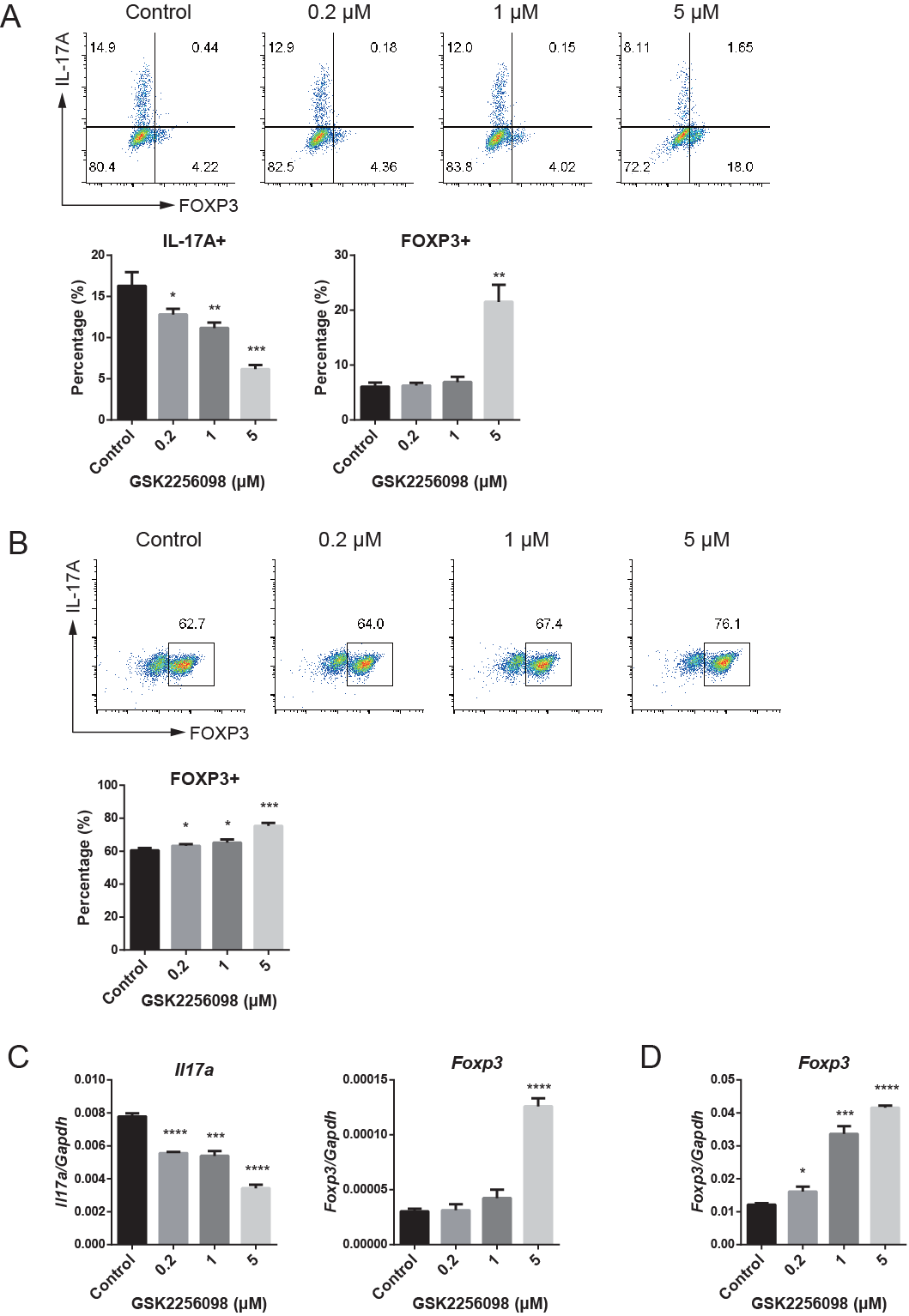


**Figure S5. FAK inhibitor GSK2256098 reduces IL-17A expression in Th17 cells, but enhances FOXP3 expression in Treg cells.**

Naïve CD4 T cells were cultured under Th17- or Treg-polarizing conditions with dose-dependent treatment of GSK2256098 for 3 days. IL-17A+ and FOXP3+ cells were measured by flow cytometry (A) and transcript levels of *Il17a* and *Foxp3* were measured by RT-qPCR (C) in Th17 cells. FOXP3+ cells were measured by flow cytometry (B) and the transcript level of *Foxp3* was measured by RT-qPCR (D) in Treg cells. Data in A to D were pooled from three independent experiments. Error bars represent the standard deviation. The significance of differences between groups was determined by Student *t* test. *, P < 0.05; **, P < 0.01; ***, P < 0.001; ****, P < 0.0001.


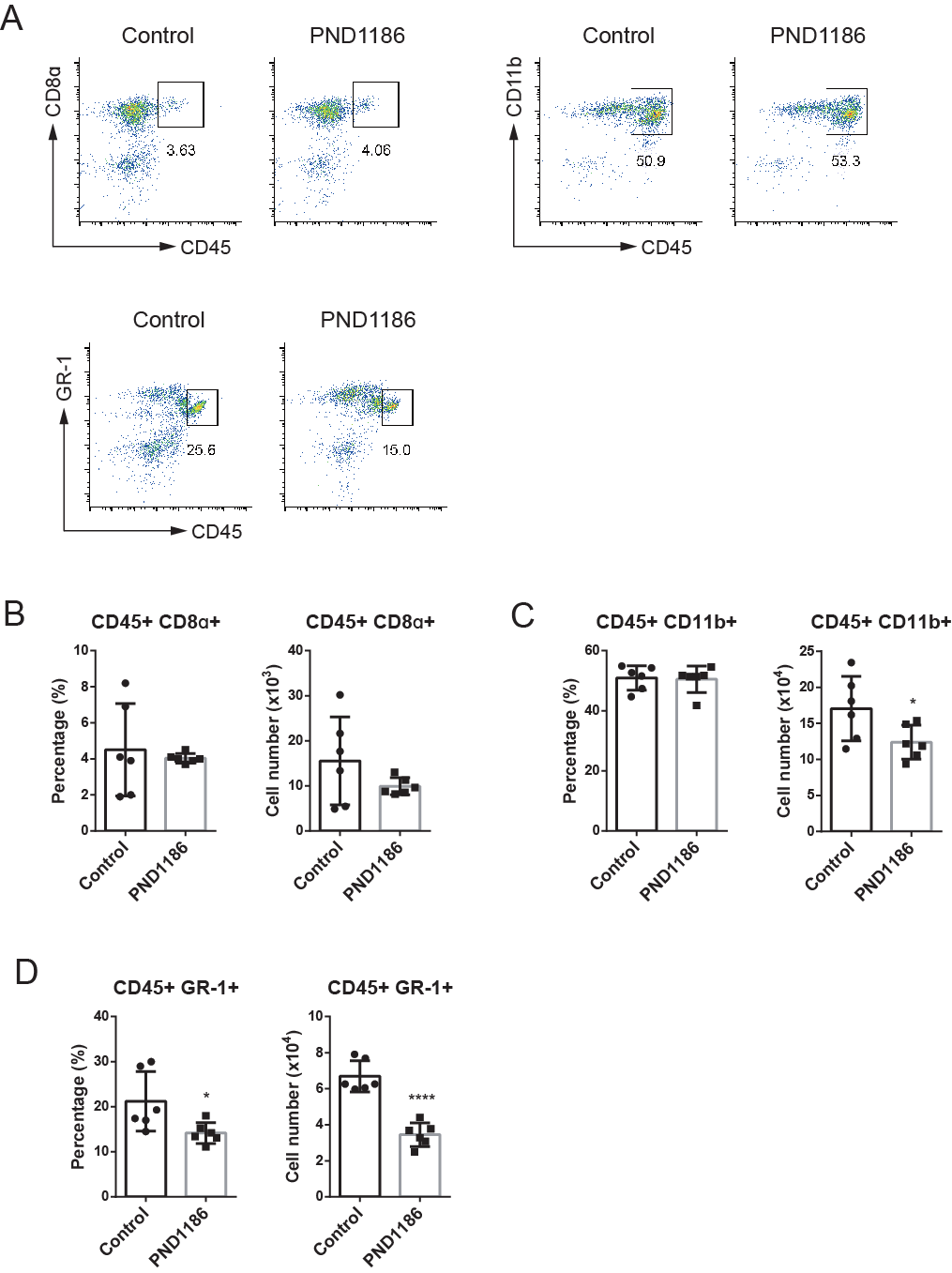


**Figure S6. PND1186 has various effects on CNS-infiltraing granulocytes, CD8 T cells, and macrophages.**

EAE was induced, and vehicle or PND1186 was treated as described in Fig. 5. (A) CD8α+ cells (left), CD11b+ cells (middle), and GR-1+ cells (right) among CNS-infiltrating CD45+ cells were measured by flow cytometry. (B to D) The percentage and absolute number of CNS-infiltrating cells were measured. (B) CD8α+ cells, (C) CD11b+ cells, and (D) GR-1+ cells. Data in B to D were pooled from six independent experiments. Error bars represent the standard deviation. The significance of differences between groups was determined by Student *t* test. *, P < 0.05; ****, P < 0.0001.

**Table S1. Primer list used for RT-qPCR**

| Primer name | Sequences (5´→3´) |
| --- | --- |

| *Fak* Forward primer | GAGTACGTCCCTATGGTGAAGG |
| --- | --- |
| *Fak* Reverse primer | CTCGATCTCTCGATGAGTGCT |
| *Gapdh* Forward primer | CAATGTGTCCGTCGTGGATCT |
| *Gapdh* Reverse primer | GTCCTCAGTGTAGCCCAAGATG |
| *Gapdh* probe | CGTGCCGCCTGGAGAAACCTGCC |
| *Il17a* Forward primer | CTCCAGAAGGCCCTCAGACTAC |
| *Il17a* Reverse primer | AGCTTTCCCTCCGCATTGACACAG |
| *Il17a* probe | TCTGGGAAGCTCAGTGCCGCCACCAGC |
| *Rorc* Forward primer | ATGAGAACACAAATTGAAGTGATCC |
| *Rorc* Reverse primer | AGTAGGCCACATTACACTGCT |
| *Il23r* Forward primer | TTCAGATGGGCATGAATGTTTCT |
| *Il23r* Reverse primer | CCAAATCCGAGCTGTTGTTCTAT |
| *Foxp3* Forward primer | CCCAGGAAAGACAGCAACCTT |
| *Foxp3* Reverse primer | TTCTCACAACCAGGCCACTTG |
| *Foxp3* probe | ATCCTACCCACTGCTGGCAAATGGAGTC |
| *Ifng* Forward primer | GGATGCATTCATGAGTATTGC |
| *Ifng* Reverse primer | CCTTTTCCGCTTCCTGAGG |
| *Ifng* probe | TTTGAGGTCAACAACCCACAGGTCCA |
| *Il4* Forward primer | AGATCATCGGCATTTTGAACG |
| *Il4* Reverse primer | TTTGGCACATCCATCTCC |
| *Il4* probe | TCACAGGAGAAGGGACGCCATGC |
| *Il2* Forward primer | TGAGCAGGATGGAGAATTACAGG |
| *Il2* Reverse primer | GTCCAAGTTCATCTTCTAGGCAC |
| *Socs3* Forward primer | AGTGCAGAGTAGTGACTAAACATTACAAGA |
| *Socs3* Reverse primer | AGCAGGCGAGTGTAGAGTCAGAGT |
| *Rela* Forward primer | AGGCTTCTGGGCCTTATGTG |
| *Rela* Reverse primer | TGCTTCTCTCGCCAGGAATAC |

**Table S2. Primer list used for ChIP assay**

| Primer name | Sequences (5´→3´) |
| --- | --- |

| *Ii17a -5* Forward primer | CAGGTATTATTCTCAGGGCTTTGG |
| --- | --- |
| *Ii17a -5* Reverse primer | TGGCAATGGTGTCTTTTCTTTG |
| *Ii17a* promoter Forward primer | CACCTCACACGAGGCACAAG |
| *Ii17a* promoter Reverse primer | ATGTTTGCGCGTCCTGATC |
| *Ii17a +10* Forward primer | GGATTAAGGGCACACGTGTTG |
| *Ii17a +10* Reverse primer | TTTCCCCACTCTGTCTTTCCA |
| *Ii17a +28* Forward primer | TCATCGGCTCCCACACAGA |
| *Ii17a +28* Reverse primer | GGCAGTACCGAAGCTGTTTCA |
| *Ii17f* promoter Forward primer | CCCACAAAGCAACACTCTTGTC |
| *Ii17f* promoter Reverse primer | ACTGCATGACCCGAAAGCA |
| *Ii17f -5* Forward primer | GCATCGCATCTTTCAAACCA |
| *Ii17f -5* Reverse primer | TTAGGATAAGCGCCCAGTGAAT |
| *Socs3* promoter Forward primer | AGTGCAGAGTAGTGACTAAACATTACAAGA |
| *Socs3* promoter Reverse primer | AGCAGGCGAGTGTAGAGTCAGAGT |
| *β-globin* Forward primer | GTTGCATTCCTCGACTGAATCCTA |
| *β-globin* Reverse primer | ACCAGTACCTGGAAGCACAATG |
| *β-globin* Probe | AAGCCACCAGCACTGTCTGCCTCAG |
